# Deep vein thrombosis and validation of the Caprini risk assessment model in Chinese orthopaedic trauma patients: a multi-center retrospective cohort study enrolling 34,893 patients

**DOI:** 10.1007/s00068-023-02265-1

**Published:** 2023-04-07

**Authors:** Xian Zhang, Anqi Hao, Yihan Lu, Weifeng Huang

**Affiliations:** 1 Department of Intensive Care Medicine, Shanghai Sixth People’s Hospital Affiliated to Shanghai Jiao Tong University School of Medicine, Shanghai, 200233 China; 2 Department of Information, Shanghai Sixth People’s Hospital Affiliated to Shanghai Jiao Tong University School of Medicine, Shanghai, 200233 China; 3 Department of Epidemiology, School of Public Health, Fudan University, Shanghai, 200032 China; 4 National Health Commission Key Laboratory of Health Technology Assessment (Fudan University), Shanghai, 200032 China

**Keywords:** Deep vein thrombosis, Orthopaedic trauma patients, Caprini risk assessment model, Chemoprophylaxis, China

## Abstract

**Background:**

The risk of venous thromboembolism among orthopaedic trauma patients is high, but prevalence of deep vein thrombosis (DVT) remains unknown. In addition, the Caprini risk assessment model (RAM) score in orthopaedic trauma patients is undetermined in previous research. This study is aimed to determine the incidence of DVT and then validate the Caprini RAM in orthopaedic trauma patients.

**Methods:**

This is a retrospective cohort study enrolling orthopaedic trauma inpatients from seven tertiary and secondary hospitals during a 3-year period (from April 1, 2018 through April 30, 2021). Caprini RAM scores were assessed by experienced nurses on admission. The patients with suspected DVT were verified through duplex ultrasonography by qualified radiologists, and then prospectively followed once a year after discharge.

**Results:**

In total, 34,893 patients were enrolled in our study. The Caprini RAM identified 45.7% of patients at low risk (Caprini score 0–2), 25.9% at medium risk (3–4), and 28.3% at high risk (5–6), highest risk (7–8), and superhigh risk (> 8). Patients with Caprini score > 5 were likely to be older, female, and with longer length of hospital stay. Moreover, 8695 patients had received ultrasonography to detect DVT. The prevalence of DVT was determined to be 19.0% [95% confidence interval (CI) 18.2–19.9%], which significantly increased with Caprini score. The area under curve of the Caprini RAM for DVT was 0.77 (95% CI 0.76–0.78) with a threshold of 4.5. Furthermore, 6108 patients who had received ultrasonography completed the follow-up. DVT patients had a hazard ratio of 1.75 (95% CI 1.11–2.76;* P* = 0.005) in the mortality, compared to non-DVT ones. Caprini scores were significantly associated with increase in the mortality [odds ratio (OR) 1.14; 95% CI 1.07–1.21;* P* < 0.001]; DVT remained an independent effect (OR 1.5; 95% CI 1.02–2.26; *P* = 0.042).

**Conclusions:**

The Caprini RAM may be valid in Chinese orthopaedic trauma patients. Prevalence of DVT and higher Caprini score were significantly associated with increased all-cause mortality among orthopaedic trauma patients after discharge. Further study is warranted to explore the causes of higher mortality in patients with DVT.

**Supplementary Information:**

The online version contains supplementary material available at 10.1007/s00068-023-02265-1.

## Introduction

Deep vein thrombosis (DVT) is a venous reflux disorder caused by abnormal condensation in deep veins that often occurs in the lower extremities. DVT may extend, develop or even detach, resulting in pulmonary embolism (PE). Venous thromboembolism (VTE), which includes deep vein thrombosis (DVT) and PE, is the most common avertable cause of hospital death, approximately affecting 5–15% of hospitalized patients for surgery or medical problem [1]. Many strategies to prevent hospital-acquired thrombosis have been developed to guide clinical decision-making targeting several populations. One of the most valued guidelines was recommended by the American College of Chest Physician [2]. In the guideline, mechanical prophylaxis such as intermittent pneumatic compression and chemoprophylaxis such as low weight molecule heparin (LWMH) were suggested as efficient approaches to prevent DVT.

In addition, several thrombosis risk assessment model (RAM) have been developed for the management of hospitalized patients at different risk. Patients at different risk levels were recommended to take appropriate preventive approaches. Commonly used models include Pauda RAM designed for medical patients [3], Greenfield RAM designed for trauma patients [4], and so on [5]. Among these models, the 2005 version Caprini RAM is the most widely used and well-validated RAM, especially for surgical patients [6]. Caprini RAM has been validated in different population, including critically ill patients [7], surgical patients [8], burn patients [9] and et al. It has been documented that the Caprini RAM could identify 25.93% of laparoscopic colorectal cancer surgical patients with superhigh risk of DVT(Caprini score, > 8) [10].

The incidence and risk profile of DVT has been determined in many hospitalized populations, including medical and surgical patients [11], cancer patients [12], pregnant women [13] and even patients with COVID-19 [14]. Orthopaedic trauma patients are a large proportion of DVT at-risk population due to reduced limb activities and potential endothelial damage. However, orthopaedic trauma consists of diverse injury types, including upper extremity injuries, foot and ankle injuries, hip fracture, and multiple injuries. Accordingly, patients with each type of injury have different incidence of DVT, varing between 1.5% in ankle fracture [15], 26.4% in femoral shaft fracture [16], 16.3% in tibia plateau fracture [17] and 35.0% in hip fracture [18]. Therefore, it may be difficult for DVT management in orthopaedic trauma patients. So far, several risk assessment models have been suggested for orthopaedic trauma patients; however, few has been validated [19]. In our study, through a large multi-center retrospective study, we aimed to show the distribution of injury sites, determine the incidence of DVT and then validate the Caprini RAM in orthopaedic trauma patients.

## Materials and methods

### Study design and anticipants

**Fig. 1:**
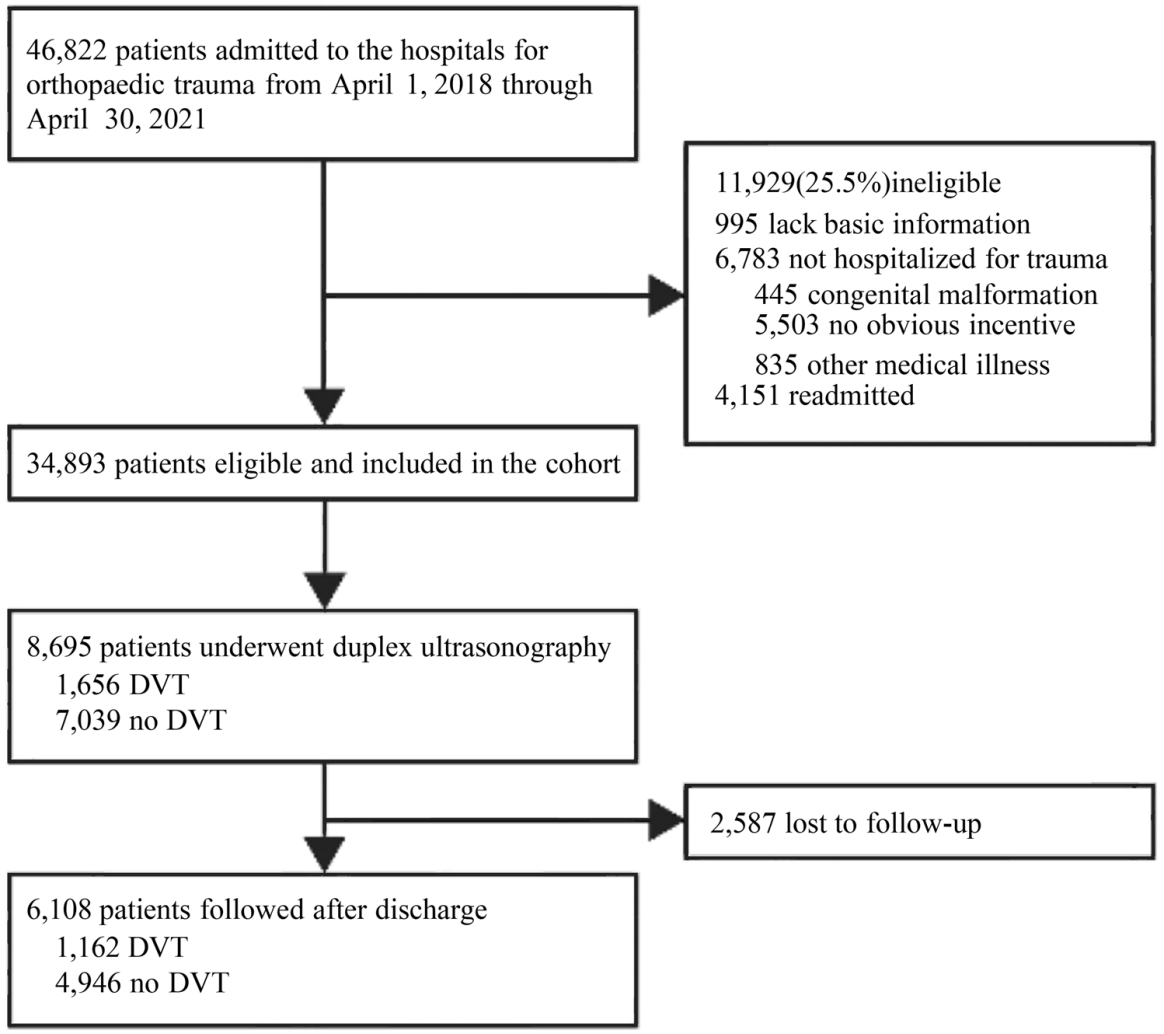
Roadmap of the patients included in the study. *DVT* deep vein thrombosis

A multi-center retrospective cohort study was conducted at seven hospitals, consisting of four tertiary hospitals and three secondary hospitals across urban and suburban areas in Shanghai, which belong to the Shanghai Sixth People’s Hospital Group with a reputation for treatment in orthopaedics in Asia. As a nationally renowned orthopedic treatment hospital, our patients come from all over the country. Patients were collected from the orthopaedic trauma wards in each hospital. Patients didn’t suffer injuries, or were admitted due to other major complaints, such as congenital defect or non-traumatic osteonecrosis of the femoral head, were excluded. Therefore, from April 1, 2018, through April 30, 2021, a total of 34,893 orthopaedic trauma patients were included in this study (Fig. 1).

Information including sex, age, height, weight, systolic blood pressure (SBP), diastolic pressure (DBP), and heart rate were examined and recorded by qualified nurses in hospital information system. Major complaints and major injury site were recorded by orthopedists. Date of admission, examination and discharge, thromboprophylaxis prescribing regimens including anticoagulation type and dose, date, and duration were directly extracted via hospital information system.

### Caprini RAM assessment

The 2005 Caprini RAM questionnaire was used to calculate risk score for all the patients admitted into orthopaedic trauma wards [5]. Caprini score was obtained by experienced nurses filling in the questionnaire during face-to-face interviews with the patients once their condition was stable. Then the score was recorded in hospital information system.

### Determination of DVT

DVT includes acute thrombosis of lower-extremity veins and up-extremity veins. As currently there is no formal screening protocol available, investigation for DVT was at the discretion of orthopedists [7]. Routinely, orthopedists examined the patients for signs and symptoms of DVT, including leg oedema and prominent veins; tenderness along the distribution of the deep venous system; calf swelling (circumference at least 3 cm greater than the other calf, measured 10 cm below tibial tuberosity) [20]. Combining the suspected clinical symptoms with Caprini RAM assessment, orthopedists decided a total of 8695 orthopaedic trauma patients to receive subsequent duplex ultrasonography (Fig. 1). DVT was determined with ultrasound imaging by qualified radiologists.

### Follow-up study

Follow-up study was conducted on the patients who had received duplex ultrasonography during 2019–2022 through an artificial intelligence calling system that is developed by the Department of Information of the Shanghai Sixth People’s Hospital. The system consists of application server, data server, telephone lines, Internet Protocol Private Branch Exchange (IP-PBX), and web server. The follow-up phone call was automatically performed once a year on the patients after discharge. In total, 6108 patients completed the follow-up (Fig. 1; Supplementary Table 1). Death and date of death were recorded.

### Statistical analysis

Patient characteristics were described and compared stratified by Caprini RAM score and DVT. Continuous variables were presented as mean ± standard deviation (SD) and then compared using a *t *test. Categorical variables were displayed with frequencies (percentages) and then compared using Chi-square test between groups. Univariate regression examined the odds for DVT among patients at different Carpini RAM risk level, odds ratio (OR) and 95% confidence intervals (CI) were calculated. Prevalence of DVT stratified by Caprini risk level was further compared. Receiver operating characteristic curve (ROC) was used to examine the predictiveness of the Caprini RAM.

In the follow-up, all-cause mortality was calculated. The Kaplan Meier curve was used to compare the survival stratified by DVT occurred during patients’ admission. Log-rank test was performed to test statistical significance by calculating the hazard ratio (HR). In addition, Cox proportional hazard regression analysis was conducted to control the influence of Caprini RAM score on DVT. Patients who remained alive by the end of the follow-up study were right censored. Analyses were performed using SPSS Statistics, version 23.0 (IBM Corporation, Armonk, NY) and GraphPad Prism version 7.0a (GraphPad Software, San Diego, California). A *P* < 0.05 was considered statistically significant.

### Ethical approval

This study involved the use of existing, routinely-collected patient-level data. All data included in the study was kept confidential without personal identifiers. No data was collected independently for the study. This study was reviewed and approved by the Institutional Review Board of the Sixth People’s Hospital, Shanghai Jiao Tong University (Approval no. 2019-087).

## Results

### Caprini RAM scores among the patients

This study included a total of 34,893 orthopaedic trauma patients with a mean age of 49.8 ± 17.7 years, in which 55.4% were male. The distribution of Caprini RAM risk were determined, including 45.7% at low risk (Caprini score, 0–2), 25.9% at medium risk (3–4), 9.0% at high risk (5–6), 12.8% at highest risk (7–8), and 6.5% at superhigh risk (> 8). Patients at higher risk (≥ 5) were likely to be older, female, with longer length of hospital stay (*P* < 0.001), in which pelvis and acetabulum fractures and multiple injuries were the most common injury sites (Table 1).

**Table 1:** Characteristics of orthopaedic trauma patients

No. (%) of patients							
Characteristics	Overall (n = 34,893)	Low risk Caprini score 0–2 (n = 15,931)	Medium risk Caprini score 3–4 (n = 9049)	High risk Caprini score 5–6 (n = 3149)	Highest risk Caprini Score 7–8 (n = 4479)	Superhigh risk Caprini Score > 8 (n = 2275)	*P* value
Age, mean (SD), years	50 (18)	45 (16)	51 (16)	55 (19)	53 (17)	68 (20)	< .001
Gender							< .001
Male, n (%)	19,328 (55.4)	9388 (58.9)	4865 (53.7)	1613 (51.2)	2496 (55.7)	966 (42.5)	
Female, n (%)	15,565 (44.6)	6543 (41.1)	4194 (46.3)	1536 (48.8)	1983 (44.3)	1309 (57.5)	
Heart rate, mean (SD)	83 (9)	83 (8)	82 (8)	83 (9)	85 (11)	84 (11)	< .001
SBP, mean (SD), mmHg	129 (18)	127 (17)	129 (17)	130 (19)	128 (19)	133 (21)	< .001
DBP, mean (SD), mmHg	78 (12)	79 (12)	79 (13)	79 (12)	77 (13)	75 (13)	
BMI, mean (SD), kg/m^2^	23.7 (3.6)	23.7 (3.5)	24.0 (3.5)	23.9 (3.6)	23.7 (3.7)	22.8 (3.8)	< .001
Length of stay, median (IQR), days	5 (4–6)	5 (4–6)	5 (4–6)	6 (4–7)	6 (5–7)	6 (6–8)	< .001
Site of injury							< .001
Upper limb	13,046 (37.4)	8605 (54.0)	3768 (41.6)	617 (19.6)	38 (0.8)	18 (0.8)	
Pelvis and acetabulum	3354 (9.6)	411 (2.6)	262 (2.9)	398 (12.6)	1254 (28.0)	1029 (45.2)	
Femoral	1102 (3.2)	409 (2.6)	201 (2.2)	180 (5.7)	193 (4.3)	119 (5.2)	
Knee	5399 (15.5)	2000 (12.6)	1464 (16.2)	552 (17.5)	1104 (24.6)	279 (12.3)	
Ankle	5491 (15.7)	2453 (15.4)	1582 (17.5)	427 (13.6)	905 (20.2)	124 (5.5)	
Other lower limb	1689 (4.8)	499 (3.1)	320 (3.5)	197 (6.3)	497 (11.1)	176 (7.7)	
Injury							
Multiple trauma	4812 (13.8)	1554 (9.8)	1462 (16.1)	778 (24.7)	488 (10.9)	530 (23.3)	
DVT							< .001
DVT, n (%)	1656 (4.7)	222 (1.4)	223 (2.5)	298 (9.5)	529 (11.8)	384 (16.9)	
Non-DVT, n (%)	33,237 (95.3)	15,709 (98.6)	8836 (97.5)	2851 (90.5)	3950 (88.2)	1891 (83.1)	
Chemoprophylaxis							< .001
No chemoprophylaxis	27,064 (77.6)	14,684 (92.2)	7563 (83.5)	2088 (66.3)	2003 (44.7)	726 (31.9)	
Enoxaparin Sodium	5986 (17.2)	1020 (6.4)	1123 (12.4)	745 (23.7)	2035 (45.4)	1063 (46.7)	
Nadroparin Calcium	1843 (5.3)	227 (1.4)	373 (4.1)	316 (10.0)	441 (9.8)	486 (21.4)	

*SD* standard deviation, *SBP* systolic blood pressure, *DBP* diastolic blood pressure, *BMI* body mass index, *IQR* interquartile range, *DVT* deep vein thrombosis

The proportion of patients who received chemoprophylaxis increased with Caprini RAM score (*P* < 0.001). Patients at superhigh risk (68.1%) were more likely to receive low weight molecule heparin (LWMH) than those at low risk (7.8%) (Table 1). Moreover, administration of Enoxaparin Sodium (17.2%) was more common than Nadroparin Calcium (5.3%) in all risk groups.

### Determination of DVT in the patients receiving ultrasonography

A total of 8695 patients with suspected DVT had received duplex ultrasonography. Among them, 1656 patients were diagnosed with DVT, which was 19.0% (95% CI 18.2–19.9%). Patients with DVT were mostly female (53.7%), with a mean (SD) age of 62.7 (16.5) years old. Mean (SD) time to DVT was 2.2 (3.7) days after admission to hospital. Between the patients with and without DVT, age, sex, SBP on admission, length of hospital stay, major injury sites showed significant differences (Table 2).

**Table 2:** Characteristics of DVT and non-DVT patient

Characteristics	DVT (n = 1656)	Non-DVT (n = 7039)	*P* value
Age, mean (SD), y	63 (17)	54 (19)	< .001
Gender			< .001
Male, n (%)	766 (46.3)	3857 (54.8)	
Female, n (%)	890 (53.7)	3182 (45.2)	
Heart rate, mean (SD)	83 (9)	83 (9)	0.681
SBP, mean (SD), mmHg	133 (20)	130 (19)	< .001
DBP, mean (SD), mmHg	76 (13)	78 (12)	< .001
BMI, mean (SD), kg/m^2^	23.8 (3.5)	23.6 (3.7)	0.015
Caprini RAM score			< .001
mean (SD)	6 (3)	5 (3)	
0–2, n (%)	222 (13.4)	1595 (22.7)	
3–4, n (%)	223 (13.5)	1529 (21.7)	
5–6, n (%)	298 (18.0)	723 (10.3)	
7–8, n (%)	529 (31.9)	2231 (31.7)	
> 8, n (%)	384 (23.2)	961 (13.7)	
Length of stay, median (IQR), d	6 (6–9)	6 (5–7)	< .001
Chemoprophylaxis			0.531
No, n (%)	839 (50.7)	3615 (51.4)	
Enoxaparin Sodium, n (%)	681 (41.1)	2803 (39.8)	
Nadroparin Calcium, n (%)	136 (8.2)	621 (8.8)	
Site of major injury			< .001
Upper limb	7 (0.4)	60 (0.9)	
Pelvis and acetabulum	500 (30.2)	1839 (26.1)	
Femoral	144 (8.7)	287 (4.1)	
Knee	480 (29.0)	1937 (27.5)	
Ankle	212 (12.8)	1634 (23.3)	
Other lower limb injury	159 (9.6)	666 (9.5)	
Multiple Trauma	154 (9.3)	607 (8.6)	

*DVT* deep vein thrombosis, *SD* standard deviation, *SBP* systolic blood pressure, *DBP* diastolic blood pressure, *BMI* body mass index, *IQR* interquartile range

With a mean Caprini RAM score of 6 ± 3, patients with DVT was mostly categorized as highest risk (31.9%) and superhigh risk (23.2%) (Table 2). The overall prevalence of DVT significantly increased with Caprini RAM score (*P* < 0.001) (Table 1). Compared with the patients at low risk, those at medium risk (OR, 1.79; 95% CI 1.48–2.15; *P* < 0.001), high risk (OR, 7.40; 95% CI 6.19–8.84; *P* < 0.001), highest risk (OR, 9.48; 95% CI 8.07–11.13; *P* < 0.001), and superhigh risk (OR, 14.37; 95% CI 12.10–17.10; *P* < 0.001) had increased risk of developing DVT. We examined the predictiveness of the Caprini RAM for DVT. The area under curve (AUC) showed the value of 0.77 (95% CI 0.76–0.78; *P* < 0.001) with a threshold of 4.5 by Youden index.

### Follow-up of the patients with and without DVT

**Fig. 2:**
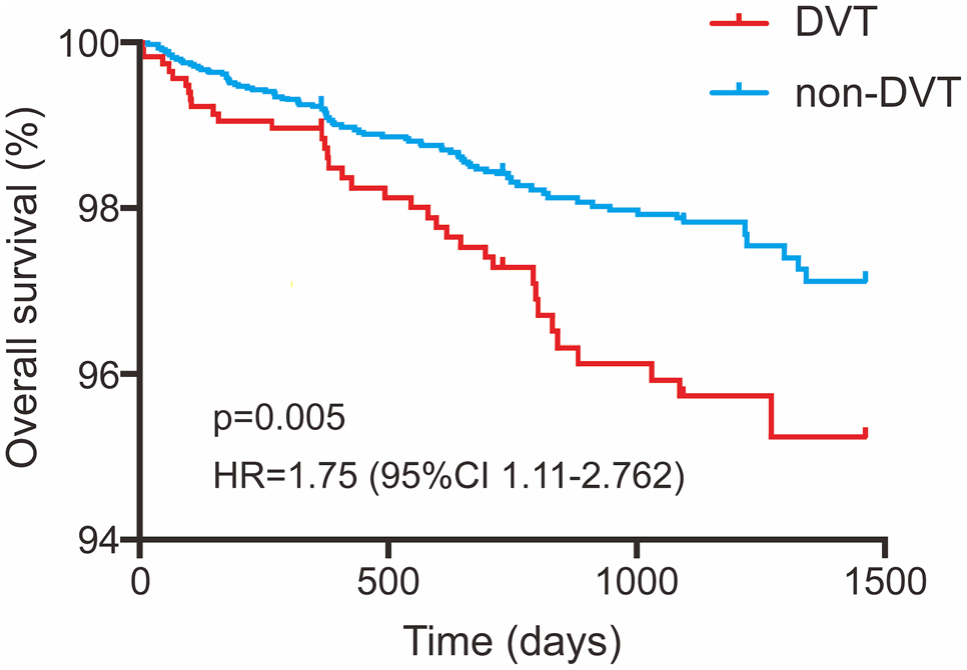
Overall survival between orthopaedic trauma patients with and without deep vein thrombosis (DVT). *HR* hazard ratio

Among 8695 patients who had received duplex ultrasonography, 6108 ones (1162 DVT and 4946 non-DVT) were followed after discharge. The characteristics of follow-up patients and lost to follow-up were presented in Supplementary Table 2. A total of 119 deaths were recorded in the follow-up and then the all-cause mortality was determined to be 1.9% (95% CI 1.6–2.3), with a median survival day of 382 (interquartile range [IQR], 166–696). In the Kaplan–Meier survival analysis, there was statistical significance between the DVT and non-DVT groups (*P* = 0.005; HR, 1.75; 95% CI 1.11–2.76) (Fig. 2). Using Cox regression analysis, we further assessed the influence of Caprini RAM score on survival. Caprini score was significantly associated with increase in the mortality (OR, 1.14; 95% CI 1.07–1.21;* P* < 0.001), while DVT also showed an independent effect (OR, 1.50; 95% CI 1.02–2.26; *P* = 0.042).

## Discussion

We retrospectively determined the prevalence of DVT and systematically validated the application of Caprini RAM among Chinese orthopaedic trauma patients. It has been documented that the AUC of Caprini RAM was 0.83 among patients with ankle fracture (n = 548) and hip fracture (n = 300) [15], compared to 0.77 in our study. However, inclusion of a total of 34,893 patients over three years among seven hospitals may allow us to present a real world scenario illustrating the distribution of injury sites, Caprini scores and prevalence of DVT across urban and suburban Shanghai. In our study, we validated Caprini RAM at each risk, and observed that prevalence of DVT increased significantly with Caprini score. It was 2.5% among patients at medium risk (Caprini score, 3–4), whereas 9.5% at high risk (Caprini score, 5–6), which was consistent with the threshold of 4.5 in the ROC. Our findings indicated that patients with Caprini score ≥ 5 should be routinely recommended chemoprophylaxis for prevention of DVT if they are not at high risk for major bleeding complications.

In our study, the prevalence of DVT was determined to be 19.0% among orthopaedic trauma patients receiving ultrasonography; furthermore, it was estimated to be 4.7% in the whole population of 34,893 patients, which was similar to the findings elsewhere [21–25]. Currently, the identification of asymptomatic DVT remains a challenge. Screening thrombosis among all orthopaedic patients is not cost-effective, therefore the judgement and credentials of physicians is particularly important [26–28]. In the *CHEST* guidelines, prevention of VTE in major trauma patients and in orthopaedic surgery patients were described separately, which may cause ambiguity for orthopedists to follow [29, 30]. As orthopaedic trauma patients have different clinical features compared with other orthopaedic patients like patients undergoing total hip arthroplasty or total knee arthroplasty [31], we suggested separately listing DVT prevention criteria for orthopaedic trauma patients, which may facilitate the prevention and management of DVT. In addition, we recommended employing Caprini RAM to identify risk levels of orthopaedic trauma patients, and determining prophylaxis accordingly.

Moreover, we conducted the follow-up of the patients receiving ultrasonography to determine the all-cause mortality. Prevalence of DVT and higher Caprini RAM score were significantly associated with increase in the mortality, suggesting a worse prognosis. Few studies have been reported to reveal mortality among orthopaedic trauma patients with and without DVT; however, the association between DVT and mortality has been illuminated among other patients. In patients with advanced cancer or COVID-19, prevalence of DVT was not correlated to the survival [20, 32]. In patients treated with immune checkpoint inhibitor, occurrence of VTE was significantly associated with increased mortality (transition hazard-ratio, 3.09; 95% CI 2.07–4.60) [33]. In addition, in medical inpatients, Caprini RAM score was correlated with in-hospital and 6-month mortality [34]. These results suggested that association between DVT and mortality remained uncertain, which warrants further validation in diverse populations.

The effects of chemoprophylaxis on DVT have been validated [35–37]. In our hospitals, two kinds of LWMH, Enoxaparin Sodium and Nadroparin Calcium, have been used for DVT prevention. However, we did not identify significant difference in the chemoprophylaxis between the DVT and non-DVT patients in our study. So far, there have remained controversial effects among orthopaedic trauma patients with different types of injury. In an American study, patients who received direct oral anticoagulants were less likely to develop DVT compared with LMWH (1.8% vs 6.9%, *P* < 0.01) in non-operative pelvic fractures patients [38]. In a meta-analysis covering eight studies targeting adults undergoing knee arthroscopy, administration of LMWH resulted in little to no difference in the incidence of PE or symptomatic DVT; in addition, it might reduce the risk of asymptomatic DVT [39]. Therefore, further study is warranted on the best practice of DVT chemoprophylaxis.

There were several limitations in our study. This is a retrospective study, in which we used routinely collected data. Data quality is a major limitation as clinical data often has more missing information than actively collected data. However, routinely collected data has enabled us to report on a very large population of patients. Furthermore, examination of ultrasonography was only conducted in the orthopaedic trauma patients who were at higher risk for DVT, which was 24.9% of all the patients. It absolutely overestimated the prevalence of DVT among orthopaedic trauma patients. Another limitation was that in the follow-up, only death and date of death have been recorded by an artificial intelligence calling system, without detailed information of death.The percentages of PE was not described and whether DVT itself was associated with mortality or not could not be determined.In future studies, we will make more detailed statistics on the causes of death and PE. In addition, compared to lost-to-follow-up, follow-up patients were likely to have more severe conditions and at higher risk, which may cause an overestimated mortality.

In conclusion, 28.3% of orthopaedic trauma patients in Shanghai were categorized as high risk, highest risk, and superhigh risk by Caprini RAM. The prevalence of DVT was 19.0% among the patients receiving ultrasonography, which significantly increased with Caprini score; furthermore, it was estimated to be 4.7% among all orthopaedic trauma patients. The Caprini RAM may be valid in Chinese orthopaedic trauma patients. In addition, prevalence of DVT and higher Caprini score were significantly associated with increased all-cause mortality among orthopaedic trauma patients after discharge.

## Supplementary Information

Below is the link to the electronic supplementary material.

Supplementary file1 (PDF 47 KB)

Supplementary file2 (PDF 116 KB)
