# Supplementary material for: Deep vein thrombosis and validation of the Caprini risk assessment model in Chinese orthopaedic trauma patients: a multi-center retrospective cohort study enrolling 34,893 patients
Source: Eur J Trauma Emerg Surg. 2023 Apr 7;49(4):1863–71. doi: 10.1007/s00068-023-02265-1 (PMC10079483; doi:10.1007/s00068-023-02265-1)
Supplement: Supplementary file 1 — Supplementary file1 (PDF 47 KB) [file 68_2023_2265_MOESM1_ESM.pdf]

Supplementary Table 1 Patients who had completed the follow-up in each year

Year of discharge	Year of follow-up			
	**2019**	**2020**	**2021**	**2022**
**2018**	1676 (75.5%)	1249 (56.2%)	997 (44.9%)	898 (40.4%)
**2019**		2218 (69.0%)	1739 (54.1%)	1550 (48.2%)
**2020**			1831 (71.2%)	1415 (55.1%)
**2021**				383 (55.5%)
