# Supplementary material for: Deep vein thrombosis and validation of the Caprini risk assessment model in Chinese orthopaedic trauma patients: a multi-center retrospective cohort study enrolling 34,893 patients
Source: Eur J Trauma Emerg Surg. 2023 Apr 7;49(4):1863–71. doi: 10.1007/s00068-023-02265-1 (PMC10079483; doi:10.1007/s00068-023-02265-1)
Supplement: Supplementary file 2 — Supplementary file2 (PDF 116 KB) [file 68_2023_2265_MOESM2_ESM.pdf]

Supplementary Table 2 Characteristics of follow-up and loss-to-follow-up patients

Characteristics	Loss to follow-up (n=4449)	Follow-up (n=4246)	*P* Value
Age, mean (SD), y	55 (18)	57 (19)	0.001
Gender			<.001
Male, n (%)	2462 (55.3)	2161 (50.9)	
Female, n (%)	1987 (44.7)	2085 (49.1)	
DVT			0.051
Yes, n (%)	883 (19.8)	773 (18.2)	
No, n(%)	3566 (80.2)	3473 (81.8)	
Admission			
Heart rate, mean (SD), beats/min	83 (9)	83 (9)	0.528
SBP, mean (SD), mmHg	130 (19)	131 (19)	0.309
BMI, mean (SD), kg/m^2	23.6 (3.7)	23.6 (3.6)	0.994
Caprini RAM score			
mean (SD)	5 (3)	6 (3)	0.001
0-2, n (%)	981 (22.0)	836 (19.7)	0.008
3-4, n (%)	911 (20.5)	841 (19.8)	
5-6, n (%)	512 (11.5)	509 (12.0)	
7-8, n (%)	1405 (31.6)	1355 (31.9)	
>8, n (%)	640 (14.4)	705 (16.6)	
Length of stay, median (IQR), d	6 (6-8)	6 (5-8)	0.107
Thromboprophylaxis			<.001
No, n (%)	2457 (55.2)	1997 (47.0)	
Enoxaparin Sodium, n (%)	1654 (37.2)	1830 (43.1)	
Nadroparin Calcium, n (%)	338 (7.6)	419 (9.9)	
Site of major injury			0.021
Upper limb	39 (0.9)	28 (0.7)	
Pelvis and acetabulum	1179 (26.5)	1160 (27.3)	
Femoral	238 (5.3)	193 (4.5)	
Knee	1243 (27.9)	1174 (27.6)	
Ankle	971 (21.8)	884 (20.8)	
Other lower limb injury	380 (8.5)	445 (10.5)	
Multiple Trauma	399 (9.0)	362 (8.5)	

SD, standard deviation; DVT, deep vein thrombosis; SBP, systolic blood pressure; DBP, diastolic blood pressure; BMI, body mass index; IQR, interquartile range.
Follow-up population：Patients followed up in several years.
Lost to follow-up population：Patients who lost follow-up in any year.
